# Recent Progress of Rational Modified Nanocarriers for Cytosolic Protein Delivery

**DOI:** 10.3390/pharmaceutics15061610

**Published:** 2023-05-29

**Authors:** Xiao He, Su Xiong, Yansun Sun, Min Zhong, Nianting Xiao, Ziwei Zhou, Ting Wang, Yaqin Tang, Jing Xie

**Affiliations:** 1 Chongqing Key Laboratory of Medicinal Chemistry and Molecular Pharmacology, Chongqing University of Technology, Chongqing 400054, China; 2 Center for Cell and Gene Circuit Design, CAS Key Laboratory of Quantitative Engineering Biology, Shenzhen Institute of Synthetic Biology, Shenzhen Institutes of Advanced Technology, Chinese Academy of Sciences, Shenzhen 518055, China; 3 Department of Geriatrics, The Shenzhen Hospital of Peking University, Shenzhen 518036, China

**Keywords:** protein therapy, cytosolic protein delivery, nanocarriers, penetrating peptide, cell-penetrating poly(disulfide)s

## Abstract

Therapeutic proteins garnered significant attention in the field of disease treatment. In comparison to small molecule drugs, protein therapies offer distinct advantages, including high potency, specificity, low toxicity, and reduced carcinogenicity, even at minimal concentrations. However, the full potential of protein therapy is limited by inherent challenges such as large molecular size, delicate tertiary structure, and poor membrane penetration, resulting in inefficient intracellular delivery into target cells. To address these challenges and enhance the clinical applications of protein therapies, various protein-loaded nanocarriers with tailored modifications were developed, including liposomes, exosomes, polymeric nanoparticles, and nanomotors. Despite these advancements, many of these strategies encounter significant issues such as entrapment within endosomes, leading to low therapeutic efficiency. In this review, we extensively discussed diverse strategies for the rational design of nanocarriers, aiming to overcome these limitations. Additionally, we presented a forward-looking viewpoint on the innovative generation of delivery systems specifically tailored for protein-based therapies. Our intention was to offer theoretical and technical support for the development and enhancement of nanocarriers capable of facilitating cytosolic protein delivery.

## 1. Introduction

Protein is a kind of organic macromolecules composed of amino acids linked by peptide bonds, which is the material basis of life. Proteins are regarded as the important component in biological system such as cell signaling pathway, cell metabolism, gene transcription and translation, and cell division and proliferation. Protein-based therapy is a promising approach for treating diseases caused by abnormalities in key cellular proteins. This therapy offers greater specificity and potency than small-molecule drugs, as proteins often require a complex structure for specificity. Additionally, some proteins play a critical role in signaling pathways, and yet cannot be targeted by small molecules, making them “undruggable”. Protein-based therapy is, thus, an attractive option for addressing such protein–protein interactions (PPIs), as macromolecules such as proteins or peptides can effectively inhibit them. In this way, it provides a link between small-molecule inhibitors and large protein targets. Therefore, protein was the potential candidate for new drugs [1,2,3,4,5,6,7,8,9,10].

In recent years, specific proteins such as adrenocorticotrophic hormone, monoclonal antibodies, enzymes, cytokines, and peptides were developed as therapeutic agents for conditions such as anti-inflammation, cancer metastasis, indigestion diseases, and viral infections [11,12,13,14,15,16,17,18]. Notably, five out of the top ten best-selling drugs in 2021 were protein antibodies, according to statistics on top companies and drug sales [19]. Consequently, the demand for protein drugs steadily increased in the pharmaceutical market.

**Figure 1:**
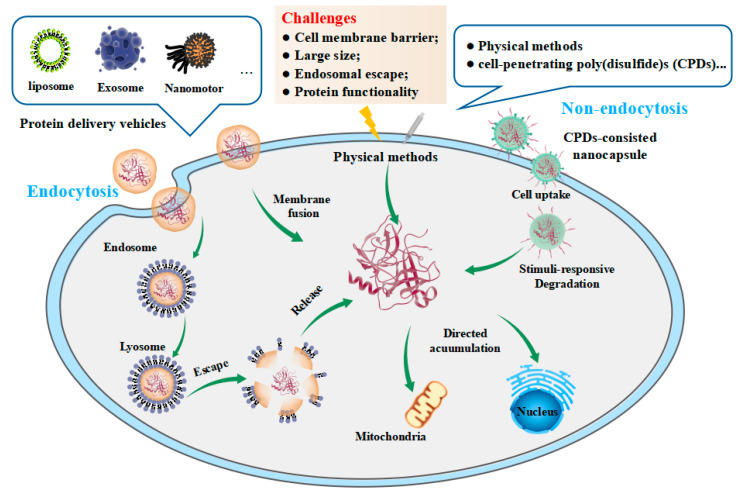
Schematic illustration of challenges in cytosolic protein delivery and currently available nanocarriers for non-endocytic delivery.

In comparison to gene drugs, protein drugs have the advantage of directly targeting the desired sites, enabling the regulation of biological activities without the complications associated with gene mutations and low therapeutic efficiency resulting from gene expression during the treatment process. However, the majority of protein drugs currently available on the market are developed based on the structure and characteristics of extracellular targets. These targets include protein receptors located on the cell membrane surface (e.g., G protein-coupled receptors, GPCRs; cluster of differentiation 4, CD4; epidermal growth factor receptor, EGFR; insulin receptor) or secreted proteins (e.g., tumor necrosis factor-alpha, TNF-α; prostaglandin 2, PG2; interleukin 4, IL-4; interferon-gamma, IFN-γ) [20,21,22,23,24,25,26,27,28]. On the other hand, the poor membrane permeability and large size of protein drugs pose challenges for their effective internalization into cells. Consequently, most biological signaling pathways or enzymes within cells are not viable drug targets due to these limitations. The importance of intracellular protein transportation extends beyond the development of protein drugs, encompassing advanced biotechnology and molecular cell biology. For instance, CRISPR/Cas9, a common gene editing tool, requires efficient transport into cells, involving zinc finger endonucleases and recombinases [29,30,31]. Consequently, there is significant value in designing novel and efficient strategies for the internalization of proteins into cells (Figure 1).

## 2. The Strategies for Cytosolic Protein Delivery

There are several challenges associated with transporting proteins into the cytosol. The primary obstacle is the cellular uptake of proteins. To enhance delivery efficiency, proteins can be tagged or conjugated with specific molecules such as cell-penetrating peptides (CPPs) [32,33,34,35,36], cell-penetrating poly(disulfide) (CPDs) [37,38,39,40,41,42,43,44], or protamine. While these approaches showed high delivery efficiency, a greater challenge lies in facilitating the process of lysosomal escape for the delivered proteins. In most cases, transported proteins are easily captured by cytosolic endosomes, which are disrupted by proteases within the endo/lysosomal structure. As a result, the amount of protein accumulated in the cytosol decreases, leading to reduced protein delivery efficiency. In recent years, significant progress was made in improving the efficiency of lysosomal escape for delivered proteins, offering great promise for further clinical applications.

Cytosolic protein delivery strategies encompass various approaches, including physical methods (Table 1), endosomolytic agents, liposomes, exosomes, nanomotors, cell-penetrating peptides, and cell poly(disulfide). Physical methods involve the puncturing of the cell membrane through techniques such as electroporation [45,46,47,48,49], nanoneedles [50,51,52,53,54], and microfluidic constrictions [55,56,57,58]. However, these methods present challenges for clinical application due to specialized instrumentation requirements. Endosomolytic agents can damage the endo/lysosomal membrane, facilitating the release of captured proteins within cells through endocytosis-mediated pathways [59,60,61,62,63]. Disruption of endosomes can be achieved by membrane-disturbing agents such as pH-responsive peptides and polymers modified with lipids or aromatic structures. These molecules exhibit unique properties that change their conformation or morphology in the acidic lysosomal space, leading to lysis of the endo/lysosomal membrane. For example, Futaki et al. reported the transportation of antibodies into cells, followed by their release in the cytosol using pH-sensitive peptides. By grafting one to two glutamic acid groups onto the hydrophobic region of cationic peptides, novel endosomolytic peptides were developed. In the endocytosis pathway, the pH-sensitive groups can become protonated due to the low pH in lysosomes, inducing lysosomal membrane disruption and the subsequent release of trapped proteins from lysosomal contents [59,64,65].

Other strategies for cytosolic protein delivery involve protein carriers such as liposomes, exosomes, nanomotors, and organic polymers [66,67,68,69,70,71,72,73,74]. These materials typically load proteins through non-covalent interactions or covalent conjugation. Proteins, being biological macromolecules, possess specific charge characteristics that can be altered by changes in solution pH, as well as three-dimensional structures with significant sizes. Although only a few sites on proteins can be modified with delivery vectors, complexes formed by non-covalent interactions between proteins and vectors may disintegrate, releasing native proteins in response to specific stimuli under certain physiological conditions [75]. The disintegration of these complexes poses a challenge to achieving high intracellular delivery efficiency of proteins in the extracellular environment. To address this, chemical modifications (such as aconitic acid or phenylboronic acid) or genetic fusion conjugation (using anionic GFP or polyglutamate) were applied to enhance the interaction between proteins and vectors [76].

The attachment of membrane-penetrating groups to the original protein can promote the permeation of cargo proteins and, then, entry into the cytosol. The generally applied functional membrane-permeated groups contain cell-penetrating peptides (CPP) [77,78,79], cell-penetrating poly(disulfide) (CPD), and the transduction domain [80]. It is the general strategy to construct the conjugates of proteins with CPP or CPD by non-covalent and covalent integration. For example, arginine-enriched CPPs could combine with cargo proteins and, subsequently, penetrate into the cytosol via electrostatic interaction with negatively charged membrane. Additionally, proteins can also be grated onto the CPDs via electrostatic interaction or covalent binding, and then improve the intracellular protein delivery with thiol-mediated endocytosis. These strategies demand the application of chemical modification or genetic fusion on the original protein, which may have a negative effect on the bioactivity and functionality of protein. For instance, the proteins fused with transduction-promoted CPPs have poor serum stability and low endo/lysosomal escape efficiency. To solve this problem, dynamic covalent linkers are applied to combine the original proteins with cell-penetrating groups. The novel conjugates of protein and polymer have the ability to release the cargo under special conditions such as lysosomal acid, protease, and glutathione (GSH) [39]. In this review, we desire to summarize the recent evolution of cytosolic protein delivery strategies, focusing on the representative cases of different materials and the novel strategies for intracellular protein delivery to supplement the previous researches. So, this perspective mainly contains fields of liposome-based carriers, nanomotor, exosome-based nano vectors, cell-penetrating peptides-based nanocarriers, and cell-penetrating poly(sulfides)-based nanocarriers (Table 1).

**Table 1:** Summary of Different Protein Delivery Methods.

Strategies	Working Principle	Protein Cargos	Advantages	Difficulties	Ref.
Physical methods	Membrane disruption		Dose-control; Uniform intracellular delivery	Nvasive electrodes; Low cell viability; Genetic perturbation; Technical restriction	
electroporation		β-galactosidase, ProSNA ^a^, Cas9-RNP ^b^,			[81]
Nanoneedles		BMP2 ^c^			[82]
Microfluidic constrictions		Saporin, Cytochrome C, Herceptin, IgG ^d^, BSA ^e^			[83]
Endosomolytic agents	Damage the endo/lysosomal membrane, release the captured protein in cells		Good biocompatibility; Potential organ or cell targeting properties; Specificity; Better endosomes escape	Endosomal capture	
Liposomes	Membrane fusion	BSA		Protein size limitation; Only deliver negatively charged proteins;	[84]
		SNARE ^f^			[85]
		ovalbumin			[86]
Exosome	Membrane fusion	SIRPα ^g^		Complex preparation process;	[87]
		Ndfip1 ^h^			[88]
		BDNF ^i^			[89]
Nanomotor	Membrane permeation	Cas9/sgRNA complex		Toxicity; Environmental issues	[66]
		Caspase-3			[90]
Cell-penetrating peptides	Membrane fusion/transduction	LAT1 ^j^	Specific delivery; High intracellular delivery Efficiency; Low cytotoxicity	Endosomal capture; Proteolytic instability; Immunogenicity; Internalization mechanisms to be demonstrated	[91]
		TG6–protein conjugates ^k^			[92]
		Ppm1B ^l^			[93]
		cytochrome C			[94]
Cell poly(disulfide)s	Membrane fusion/transduction	β-galactosidase	Membrane permeating; Bioactive	Off-target; Metabolic barrier; Toxicity; Endosomal capture; Big size of protein	[95]
		BSA, RNaseA, Cetuximab			
		BSA, anti-MTCO2			[96]
Fluoropolymers	Membrane fusion/transduction	BSA, β-galactosidase, porin, a cyclic hendecapeptide Dopamine	Hydrophobicity; Lipophobicity; Good chemical stability; Bio-inertness; Low surface energy; Phase segregation	Toxicity; Environmental issues; Blood circulation time; Exact mechanisms of; The endocytosis and Endo/lysosomal escape processes to be demonstrated	[97]

^a^ protein spherical nucleic acids (ProSNA); ^b^ Cas9-ribonucleoprotein complex (Cas9-RNP); ^c^ bone morphogenetic protein 2 (BMP2); ^d^ immunoglobulin G (IgG); ^e^ bovine serum albumin (BSA); ^f^ soluble N-ethylmaleimide-sensitive factor attachment protein receptor (SNARE); ^g^ signal regulatory protein α (SIRPα); ^h^ L-domain-containing protein (Ndfip1); ^i^ brain derived neurotrophic factor (BDNF); ^j^ l-type amino acid transporter 1 (LAT1); ^k^ a dendritic small molecule TG6 with one rigid planar core and four flexible arms with one guanidinium on each arm; ^l^ protein phosphatase 1B (Ppm1b).

## 3. Nanocarriers-Mediated Protein Delivery

### 3.1. Liposomes

**Figure 2:**
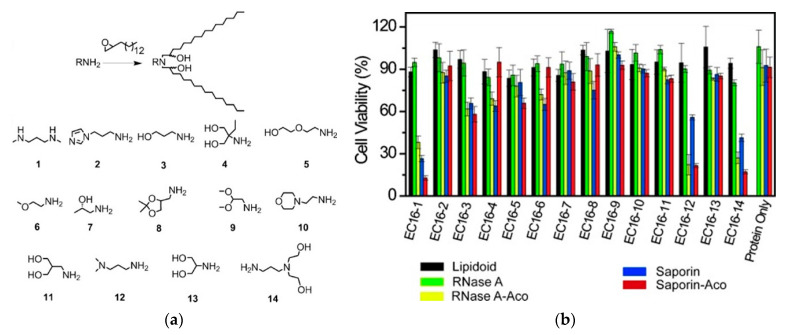
(**a**) Route of synthesis for lipidoids. The chemical structures of the library of amines used for lipidoid synthesis (the lipidoids are named EC16, for 1, 2-epoxyhexadecane, followed by the amine number); (**b**) evaluation of lipidoid-facilitated protein delivery on B16F10 cell line via a cytotoxicity assay. Black: lipidoid controls (4 mg/mL); green: RNase A (3.3 mg/mL); yellow: RNase A-Aco (3.3 mg/mL); blue: saporin (0.17 mg/mL); red: Saporin-Aco (0.17 mg/mL). Cytotoxicity was determined by MTT assay. Data are presented as mean SD (*n* = 4). Reproduced with permission from ref. [101]. Copyright 2014 WILEY-VCH Verlag GmbH & Co.KGaA, Weinheim.

Researchers successfully used liposomes to deliver therapeutic proteins such as antibodies, cytokines, and enzymes into mammalian cells. The phospholipid bilayer could retain the biological activity of proteins in the cavity, which could shield the damage to the extracellular matrix and endo/lysosomes in the cell to proteins [98,99,100]. These liposomes enter the cell through the endocytosis-mediated pathway. After the vesicles are wrapped by endo/lysosomes, therapeutic proteins can be released into the cytoplasm as a result of disruption and disruption of the endo/lysosome. Xu et al. successfully developed an efficient and safe carrier to deliver active proteins into the cytoplasm, providing ideas for protein-based therapy in the future. A novel protein delivery carrier can be synthesized by combining cationic liposome-like materials, and the method of reversible chemical modification of protein is used to improve the negatively charged density of cargoes for binding with cationic carriers. Two representative toxic proteins, ribonuclease A (RNase A) and saporin (SA), were applied to kill cancer cells via liposome-based cytosolic protein delivery in the experiment. The combinatorial liposomes can efficiently transport proteins into the cytoplasm of cancer cells and inhibit cell proliferation. The research shows that the electrostatic and hydrophobic interactions between liposomes and proteins play an important role in the nanocomposites formed by proteins and liposomes. Among them, the liposome EC16-1 protein nanoparticles can effectively inhibit cell proliferation in vitro (Figure 2) [101].

Membrane fusion liposomes are a special class of liposomes. These monolayer liposomes can quickly merge with the cell membrane and, then, transport the substances in the liposome cavity to the cytosol [102]. Csiszar et al. successfully developed a novel method for using fusion liposomes to efficiently deliver proteins. Both the positively charged carrier and negatively charged protein formed a protein-liposome complex through electrostatic and hydrophobic interaction. Liposomes can efficiently merge with the cell membrane and release intact proteins into the cytoplasm [103]. Proteins such as green fluorescent protein, RNase, saporin, and phycoerythrin can be successfully transported into mammalian cells. It is worth noting that proteins with positive charge cannot be transported into the cytoplasm through this strategy. Because positively charged proteins and cationic liposomes with similar charges repel each other, they are restricted from forming liposome-protein complexes. Subsequently, Jiang et al. successfully designed and synthesized a delivery system, which avoided the electrostatic repulsion between the protein and the bilayer phospholipid of fusion liposome [104], thus overcoming the delivery challenge of protein with positive charge. After this, cytochrome C was loaded into the cavity of mesoporous silica nanoparticles, the outer layer of which was modified with a fusion liposome bilayer. Even with the application of endocytosis inhibitors in this cell experiment, the amount of mesoporous silica nanoparticles (MSN) delivered into cells almost did not decrease, which indicated that MSNs-based protein delivery depended on the membrane fusion-mediated pathway. The results confirmed cytochrome C was successfully released in cells, leading to efficient apoptosis. However, proteins with large size are difficult to enter into cells via this system.

### 3.2. Exosomes

**Figure 3:**
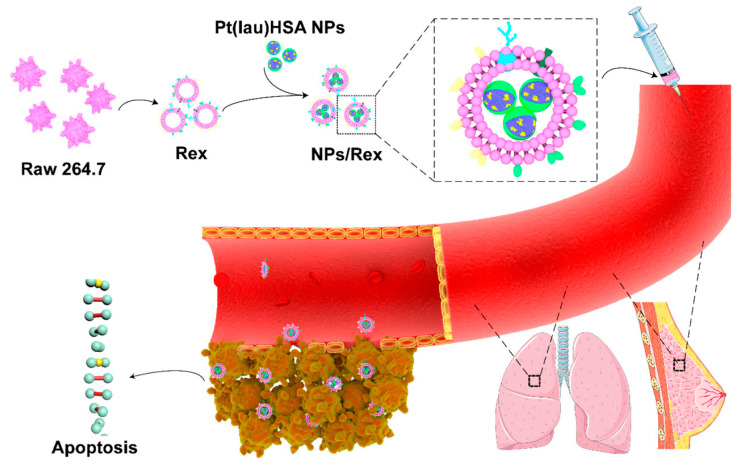
Schematic illustration of the Pt(lau)HSA NP-loaded exosome platform (NPs/Rex) for efficient chemotherapy of breast cancer. Pt(lau), HSA NPs, comprising Pt(lau), HSA, and lecithin, were coated with exosomes isolated from RAW 264.7 cells. Reproduced with permission from ref. [73]. Square symbolizes the selected part. Copyright 2019 American Chemical Society.

Exosomes are naturally secreted vesicles of several cells and tissues, which comes from cell compartments and participate in the transmission of information from one cell to another. It was proved that exosomes are used to encapsulate and deliver foreign macromolecules into the cytoplasm [105]. For example, Wu et al. first prepared the prodrug of cisplatin with lauric acid. Human serum protein and lecithin were mixed together in a certain proportion to prepare nanoparticles by nano-precipitation method, and then accurately characterized by fluorescence spectrum. Recent studies reported that macrophages can preferentially target the lesion site of breast cancer, and the exosomes can be secreted by mouse-derived mononuclear macrophages RAW 264.7, and the secreted exosomes can wrap the above prepared nanoparticles [73]. This high-performance delivery system, known as NPs/Rex (Figure 3), has excellent physical and chemical properties, high colloidal stability, and a redox-triggered release function. The study of cell dynamics proved that nano preparation entered the cytoplasm through multiple ways to avoid the trap of endo/lysosomes, and successfully degraded in the cytoplasm, then releasing the original drug and entering the nucleus to exert its bioactivity. Subsequently, a breast cancer cell experiment was carried out, and the results showed that the nanoformulation had a strong inhibitory effect on cell proliferation. For the application of the experiments in vivo, the exosomes wrapped with nanoparticles have good blood circulation and a strong inhibitory effect on the growth of cancer cells in the mouse breast tumor model. In recent years, Xu et al. reported an easily applicable and multifunctional method to modify exosomes, and then connect them for intracellular protein delivery [106]. The designed strategy is to use the active group azide to modify and label the active protein or glycoprotein involved in the metabolic process of secreting and synthesizing exosomes, and then modify and functionalize exosomes through biorthogonal click chemical reaction. Azide-modified exosomes connect a series of small molecules or proteins, thus effectively promoting exosomes to enter the cytoplasm. Metabolic engineering of exosomes can promote their multifunctionality through chemical modification, thus expanding the application of exosomes. At the same time, it provides a novel and powerful tool to study the multiple roles of exosomes in organisms and enhance the potential of the biopharmaceutical application of exosomes.

### 3.3. Nanomotor

Nanomotors are miniscule devices that imitate biological motors, constructed from a few nanoscale components and programmed to perform mechanical activities when exposed to certain stimuli such as light, sound, magnetism, electricity, and heat. So, nanomotors were suggested as the next generation of nanocarriers in the drug delivery due to their autonomous motion and associated mixing hydrodynamics, especially when acting collectively as a swarm [66]. In the protein delivery field, nanocarriers have great application prospects. Recently, Chen et al. published that gold nanowires driven by ultrasound can be used for the transport of oligonucleotides [107]. The nanomotor quickly punched and passed through the cell membrane and then delivered small interfering RNA (siRNA) into the cytoplasm, resulting in the obvious 94% down-regulation of the target protein. Inspired by this, Wang et al. further used ultrasonic-driven nanomotors to transport caspase-3 into the cytosol of gastric adenocarcinoma cells. The outer layer of gold nanowires was modified with a pH-sensitive polymer loaded with caspase-3. The outer polymer can maintain the native caspase-3 in the extracellular acidic environment without premature release and degradation of cargoes. Under ultrasound stimulation, these gold nanowires can be driven and rapidly migrate, which promotes cell uptake of protein cargo. In the cytoplasmic environment, the polymer can be rapidly degraded, and then release caspase-3, which can induce apoptosis of 80% of cancer cells within a few minutes. The similar nanomotor was able to deliver Cas9/sgRNA complex for the knockout experiment of green fluorescent protein. After 2 h of cell culture, the nanomotor loaded with Cas9 sgRNA led to the down-regulation of 80% green fluorescent protein [66]. In addition, Sánchez et al. designed an enzyme-powered nanomotors, which can be disrupted by laser irradiation. The urease-powered motion and swarm behavior improve translational movement compared to the passive diffusion of nanocarriers. Meanwhile, the synergistic effect of active motion and mechanical disruption (light-triggered nanobubbles) of a biological barrier represents a clear advantage for the improvement of therapies [108].

## 4. Non-Endocytic Protein Delivery

### 4.1. Physical Methods

Physical methods are considered the most straightforward and traditional manners to accomplish the cytosolic delivery of proteins. A few strategies were developed, such as microinjection and electroporation [109,110,111]. The main mechanism of these methods is to destroy the target cell membrane in vitro applications, so that the therapeutic proteins can be delivered into the cytosol with immediate bioavailability. For instance, Park et al. developed a novel microneedle system that involves physically attaching active pharmaceutical ingredients particles to the biocompatible adhesive surface of the microneedles named particle-attached microneedles, which can provide a wide range of applications for dosing drugs and vaccines [112]. In addition, various physical methods were used in the field of protein delivery. Notwithstanding, these physical techniques also pose some challenges, such as low-efficiency of throughput, disruption to cells, and requiring specialized instrumentation to physically puncture cell membranes, which limit their utility for largescale and pharmaceutical applications. Moreover, for human diseases, the volume of tissues that can be dosed by physical methods is very limited, and such manners can also lead to the outsourcing of cell contents, leading to corresponding biosafety issues [113].

### 4.2. CPP-Modified Protein Delivery

Cell-penetrating peptides (CPPs), regarded as protein transduction domain, are short positively charged peptides composed of 5–30 amino acids, which can pass through the cell membrane, then achieve the delivery and release of the cargo into the cytoplasm under physiological pH condition. A wide range of CPPs were developed and designed with arginine-enriched CPPs, with polyarginines and HIV-1 TAT being the most widely used for research. Recently, CPPs were always the subject of a heated debate because of their high intracellular delivery efficiency and low cytotoxicity [114,115]. Due to the ability of these peptides to penetrate into the cell membrane, they are regarded as a promising and potential tool for internalization into cell. In order to study why CPPs with positive charge can penetrate across cell membranes, a lot of relative studies were conducted. The mechanism for CPPs-mediated cellular uptake of cargos was a hot topic of intense investigation [116]. Initial studies indicated that CPPs were translocated across cellular membrane via direct penetration mechanism, which avoid the endocytosis and the participation of sepical receptors. However, in 2003, Richard et al. highlighted the likelihood of errors in the results of direct penetration experiment. Since then, research on the active transportation of CPPs was conducted, with the majority of both older and more recent studies suggesting endocytosis as the primary route of entry for CPPs into cells [117]. The cellular uptake mechanism of CPPs is debated. Thus, a progression of results indicated that cell-penetrating peptides are taken up by cells through energy-independent direct penetration and energy-promoted endocytosis, making these CPPs convenient for entering the cytosol of mammalian cells through a variety of mechanisms. The endocytosis efficiency was attributed to the cell penetrating peptide itself (peptide length and physicochemical properties), as well as the conditions of cell internalization (peptide concentration, lipid components and membrane zeta potential). Changes in these elements can lead to the transformation of the main internalization mechanisms. The difference between CPP-cargo and CPP is that the physiochemical property of the cargo also affects the cell uptake mechanism. Therefore, it is still difficult to predict the delivery efficiency of this specific CPP–cargo conjugation [118].

The transportation of essential small molecules such as amino acids, sugars, and ions is carried out by integral membrane protein pumps and channels. For macromolecules, the different system is needed to pass through cellular membrane, which requires energy. Endocytosis is an active process in which macromolecules are taken up into the cell in vesicles that are separated from the plasm membrane and involves two stages: endocytosis uptake and endo/lysosomal escape. Arginine-enriched peptide has the ability to penetrate across the biological cell membrane and reach the cytosol and nucleus in the direct penetration manner. The experimental results indicated that the cytosolic delivery of CPPs still happen at low temperature. The energy-mediated endocytosis of CPPs would be prohibited below 4 ℃ conditions. Therefore, the CPP direct penetration was the main way of cell internalization. The guanidine grafted onto the CPPs can bind with the anions on the surface of the cell via electrostatic interaction and hydrogen bonds. The non-covalent interaction effects induced the CPP aggregation on the cell surface, which was convenient for the endocytosis. Once the cell penetrating peptides are transported into the cytoplasm via forming complexes with membrane phospholipids, they subsequently release the CPP from complexes through replacing lipids with the intracellular anions. Cardoso’s group confirmed that guanidine groups can bind with fatty acid on the cell surface by non-covalent interaction, followed by enhancing the cell internalization of arginine-enriched peptides [119]. Transmembrane pH gradient variation is a driving force, which can promote the cellular uptake of polypeptides. In addition, the energy-independent direct penetration was also related to various factors including peptide density, sequence and the lipid component. If the direct penetration efficiency is too slow, the competitive endocytosis-mediated mechanism begins to operate, and the CPPs would be intercepted in the endo/lysosome, while direct penetration into cytoplasm of cells occurs in some cases, mainly at a high concentration of CPPs. Generally, CPPs with high concentration promote the entry of cargos into the cytosol with direct penetration manner. It is accepted that most CPPs and CPP-cargo complexes are taken up by cells through endocytosis pathway.

Therapeutic agents should be delivered into the cytosol of cells to exert their bioactivity. Because of the poor permeability of the cell membrane, free cargos such as proteins, peptides, and nucleic acids were restricted from entering the cell. Since CPPs have the ability to deliver different cargoes without obvious cytotoxicity, they are applied to enhancing the cytosolic delivery efficiency of relative drugs. Therefore, wide biomedical application of CPPs include anticancer, vaccines, antimicrobials, and anti-inflammation, and regarded as the vector to deliver nucleotides, peptides, and proteins. The recent studies indicated the CPPs were applied to induce anti-inflammatory effect. According to the literature, nuclear factor-κB (NF-κB) is a protein complex that has an important effect on adjusting the gene transcription related to inflammation, such as enzymes, chemokines, and cytokines. NF-κB incontrollable activation leads to different inflammatory disease includes viral infection, rheumatoid arthritis, and enteritis. In this regard, different strategies were used for improving the abnormal NF-κB protein. Wang et al. evaluated that NF-κB was inhibited by anti-inflammatory peptides termed AIP6, which has the ability of cell-penetrating properties. AIP6 can also combine with the subunits of NF-κB named p65 to adjust its bioactivity, thus achieving the desired anti-inflammatory effect [120].

**Figure 4:**
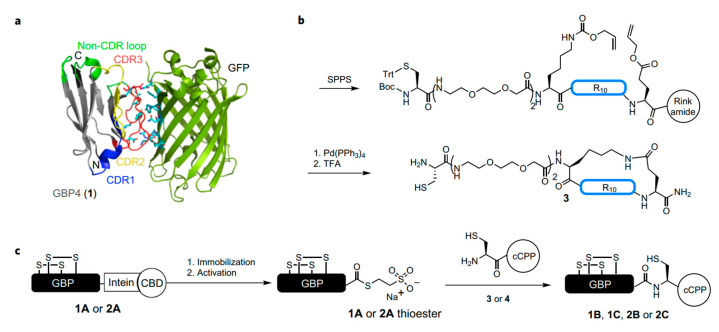
Synthesis of cell-permeable antigen-binding proteins. (**a**), X-ray structure of GBP4 (1) binding to its antigen GFP. The variable antigen-binding CDRs (complementary determining regions) 1–3 are highlighted in blue, yellow and red, respectively. The loops of the conserved nanobody framework that are the most distal to the antigen-binding interface and, thus, best for site-specific functionalization are highlighted in light green (PDB ID: 3G9A)37 and GFP is drawn in dark green. (**b**), Scheme for the synthesis of Cys–cR10 (3). Trt, trityl. (**c**), Synthetic strategy of GBP–cCPPs. The full length nanobody (*1A* or *2A*) is expressed as an intein–CBD fusion and is reacted with the Cys-containing CPPs (*3* or *4*) by MESNA-induced ligation. Comprising Pt(lau), HSA, and lecithin, these were coated with exosomes isolated from RAW 264.7 cells. Reproduced with permission from ref. [123]. Copyright 2017 Nature.

Recent studies about HIV-TAT peptides indicated that the intracellular delivery efficiency of arginine-enriched molecules is promoted with increasing structural rigidity. Cyclic TAT peptides demonstrated higher cellular internalization efficiency compared with their linear and more flexible analogue molecules [121]. To study whether CPPs can promote cellular internalization of proteins through endocytosis pathway. Hackenberger et al. designed and synthesized different ring and linear GFP–CPP conjugation with alkynyl-tagged GFP and azide modified CPPs [122]. The results indicated that the cyclic CPP–GFP conjugation could be efficiently taken up by living cells, which can enable protein to reach the cytosol and nucleus, but its linear analog could not assist the GFP to enter the cell. Depending on the promising data, this method was used to deliver monoclonal antibodies. The authors designed and developed an unified method to construct the special membrane-penetrated nanoantibodies that can be efficiently taken up by cells. Nanoantibodies are applied to replacing traditional monoclonal antibodies. Because of their with relatively large size, they may be difficult to penetrate across the membrane with the CPPs-assisted manner. The authors designed a GFP targeted protein, and the special site was modified with a cyclic CPPs. The experimental results show that these nanoantibodies were convenient for the entry into the cell and interacting with intracellular GFP (Figure 4). CPP-nanoantibody conjugates can tag and control antigens or ligands interacting with them. For example, two nanoantibodies fused with GFP were used to track and relocate polymer enzyme clamp (PCNA) and tumor inhibitor P53 in the nucleus. In addition, CR_10_-nanoantibody can enhance intracellular delivery of respective antigens and antigen-conjugated proteins, such as Mecp2 fused with GFP. When CR_10_ induced the redistribution of the antigen in the nucleus, and CR_10_ nanoantibody with a cleavable disulfide can be disrupted for releasing antigens and their partners by intracellular GSH stimulation. These nanoantibodies can be efficiently delivered into cells and, subsequently, interact with the GFP-PCNA protein complex to realize the track and relocation of targeted protein in the nucleus [123].

**Figure 5:**
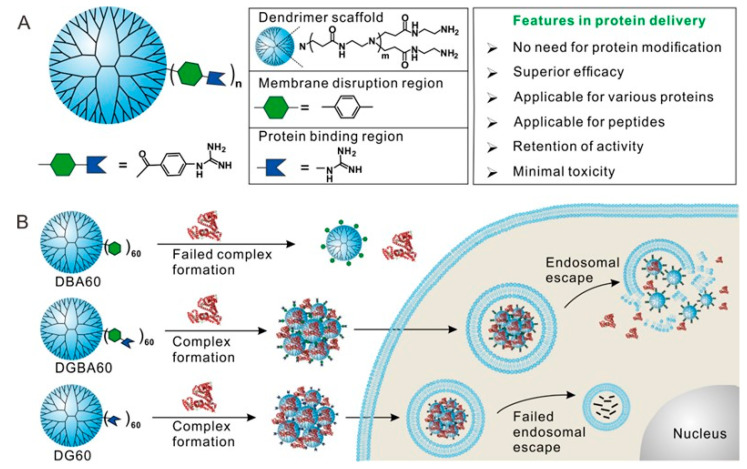
Structure of the designed polymer and its features in intracellular protein delivery (**A**). Proposed mechanism of the designed polymer and control materials in intracellular protein delivery (**B**). Reproduced with permission from ref. [128]. Copyright 2017 American Chemical Society.

In order to enhance the interacting affinity of cationic polymers and proteins, researchers choose polymers enriched with guanidine. Because guanidine can form strong electrostatic interaction and hydrogen bonds with the carboxyl groups of glutamic acid or aspartic acid in protein. The affinity between guanidine and carboxyl is stronger than that between ammonium salt and carboxyl [124,125]. Because of the special biological properties of guanidine, polymers enriched with guanidine can be used as protein adhesive, and Aida et al. used it to regulate the biological function of the active protein [126]. If highly intensive guanidine groups are modified on the polymer, the cationic polymer will significantly enhance its binding with proteins through multivalent electrostatic effects. In addition, guanidine can interact with phospholipid bilayer to promote cell uptake. Arginine-rich peptides, polymers, and nanoparticles have relatively good membrane translocation effects. For example, cell-penetrating peptides are convenient for conjugating or assisting cargoes to complete the process of intracellular delivery [127]. According to the above mentioned rational design strategies, Cheng et al. successfully developed three kinds of polymers (Figure 5). Each dendrimer contains an average of 60 guanidines, benzoic acid, and 4-guanidine benzoic acid. The last one can efficiently promote the intracellular proteins delivery in these polymers [128]. Guanidine’s were applied to combine with proteins, and guanidine linked phenyls possess an important effect in the process of endo/lysosomal escape. In addition, the guanidine–π fore among molecules or the electrostatic interaction between guanidine groups is indispensable for the stability of the complex, and it is also a key factor of protein delivery in the cytosol. Bioactive proteins including β-glucosidase and p53 can be transported via DGBA (phenyl guanidine dendrimer) into the cytosol, and then released, still maintaining the original biological activity of the protein. In addition, DGBA can also efficiently deliver bioactive peptides into the cytosol, and apoptosis-promoting peptides can efficiently enter the cytoplasm of cancer cells, inducing a large number of cancer cells to apoptosis.

Despite the potential of CPPs for protein delivery, certain limitations remain to be addressed, such as the lack of cell specificity. To overcome this, it is necessary to create delivery systems that can target tumors and be permeable to cell membranes, enabling site-specific delivery and internal trafficking. Such systems could be designed using tumor-targeted peptides, or through multi-component delivery systems that enhance the delivery and release of proteins. In addition, the positive charge of CPP is often responsible for causing extreme cytotoxicity and hindering its ability to deliver proteins. So, further research and improvement are required to explore the effects of exogenous substances on the membrane penetration mechanism of CPPs when they are connected to them, as well as the entry sites and targeting properties into the cell, metabolism, and degradation with endo/lysosomes. Additionally, in vivo studies of CPPs must be further explored to fully realize the potential of peptide-based protein delivery systems [129,130]. All in all, with in-depth research on interdisciplinary applications, CPPs will have an increasingly significant role in the treatment of animal and human diseases.

### 4.3. CPD-Based Intracellular Delivery of Native Proteins

CPDs, which are synthetic analogs of CPPs, are characterized by disulfide bonds instead of a peptide skeleton. In addition, CPDs can be grown on substrates through surface initiated ring-opening disulfide-exchange polymerization. Compared to CPPs, CPDs can enter cells and deliver cargo to the cytosol via thiol-mediated pathways without being trapped in endocytic vesicles. Furthermore, CPDs can be degraded in the cytosol by glutathione (GSH)-assisted depolymerization in a short time and have minimal cytotoxicity, making them suitable for intracellular delivery of thiol-containing or modified cargos [131,132]. The strategy of applying organic polymers to develop and prepare therapeutic agents is promising. Recently, CPDs were elaborately designed and produced. It was widely recognized as the great potential cell penetrating candidate molecules that tremendously expands the diversity of biomaterials. The designed CPDs were achieved through substrate-initiated ring opening disulfide-exchange polymerization in solution by Matle’s group in 2013 [133,134]. They conducted a series of studies, in which the cyclic tension played a role in the thiol-based cell uptake promoted by cyclodisulfide polymers. As the ring tension increased, the cell uptake efficiency of CPDs were further increased. According to the reports, carbon-sulfur–sulfur-carbon (CSSC) dihedral angle was eight degrees, leading to the obviously different uptake efficiency of CPDs by cells. The uptake efficiency enhanced by high cyclic tension was superior to that promoted by linear disulfide tension [135]. The exchange from thiol to disulfide of the membrane would affect the uptake behavior of cell [136]. So, the decrease in disulfide to thiol led to an increase in uptake efficiency. These data indicate that dynamic covalent exchange affects the uptake of CPDs by cells.

In recent years, poly(disulfide)s with positive charge were developed for intracellular gene delivery. The cationic poly(disulfide)s were explored and synthesized through Michael addition reaction of amino groups instead of disulfide-exchange polymerization on the surface of solid substrates. In less than 5 min, the CPDs were directly obtained on solid substrates under physiological pH and 25 °C conditions. The siCPD can be divided into three parts: an inducer, a monomer, and a terminator [137]. The monomer contains not only a disulfide bond for ring-opening disulfide polymerization, but also a cationic guanidine group to ensure membrane permeability. The synthesis of CPDs can be carried out rapidly at room temperature and neutral pH value. Through the application of CPDs containing fluorescent probe inducers, we found most of the simplest yet active CPDs, which, upon cultivation with cells, can reach the cytoplasm in several minutes, and then degrade rapidly to release the original biological molecules. After the depolymerization of CPD, the membrane disturbance stopped, and the toxicity was very low even at high concentration. The author further confirmed that the intracellular delivery of CPDs is not sensitive to endocytosis inhibitors, and less dependent on temperature, but is greatly affected by a thiol blocker. Hence, CPDs need to solve the two main limitations of endocytosis mediated delivery strategies: the escape of endo/lysosomes and the toxicity of biomaterials [131]. Subsequently, it was found that CPDs enhanced the efficiency of cell internalization through dynamic covalent disulfide substitution with thiol on the cell membrane, and entered the cytoplasm by avoiding the endocytosis pathway [138]. Subsequent studies indicated that even the monomer itself, or its tension-enhanced analogues, such as diselenolanes and epidithiodiketopiperazine, can enter cells with high efficiency. With the increase in protein molecular weight, most CPP-assisted delivery strategies tend to penetrate into the cell through endocytosis, and eventually be trapped by endo/lysosomes in the cell, unable to escape the fate of degradation.

The unique characteristics of CPDs polymers make them highly effective in promoting cellular uptake of cargo molecules. Recent research by Yao et al. focused on synthesizing a library of CPDs, enriching the delivery system, and expanding the application of CPDs for cell internalization of protein drugs. Their approach aims to reduce the probability of endo/lysosomal capture while preserving the bioactivity of the delivered proteins. To achieve this, protein–CPDs conjugation was designed and developed through covalent reactive modification or non-covalent interactions between proteins and CPDs.

For instance, initiators such as nitrilotriacetic acid (NTA), biotin, and tetrazine were incorporated onto CPDs polymers, which can then be incorporated into proteins modified with His, avidin, and trans-cyclooctyne (TCO) through non-covalent or covalent bonds. Antibodies can react with trans-cyclooctyne (TCO) and, subsequently, graft onto the corresponding CPDs with initiators including tetrazine via biorthogonal chemical reactions. These multi-functional CPDs-based methods gained recognition for their application in cytosolic protein delivery due to their special and potential properties: (1) simplified modification: biorthogonal modifications can be easily completed by simply mixing the components in aqueous solutions under mild conditions. (2) Enhanced delivery efficiency: engineered proteins and antibodies demonstrate improved cellular delivery efficiency using CPDs-based methods, primarily due to direct translocation instead of relying on common endocytosis pathways. (3) Improved biocompatibility: CPDs exhibit excellent biocompatibility, making them suitable for protein delivery applications.

In a related study, Matile et al. developed a potential protein delivery strategy based on the non-covalent affinity effect between biotin and streptavidin. However, methods involving chemical or gene-engineered modifications may impact the structure and bioactivity of proteins [136]. As an alternative, a CPD-coated nanocapsule composed of biodegradable organosilicon was successfully designed. This nanocapsule contains therapeutic antibodies, fluorescence dyes, and quenchers, allowing their delivery into the cytosol of cells while evading lysosomal capture and enabling on-demand release of native proteins under hypoxic-responsive conditions. The nanocapsule’s surface is further modified with mitochondria-targeted triphenylphosphonium (TPP) for precise disruption of the mitochondria. It is important to note that robust conditions used during nanocapsule preparation can potentially damage the bioactivity of the cargo proteins.

**Figure 6:**
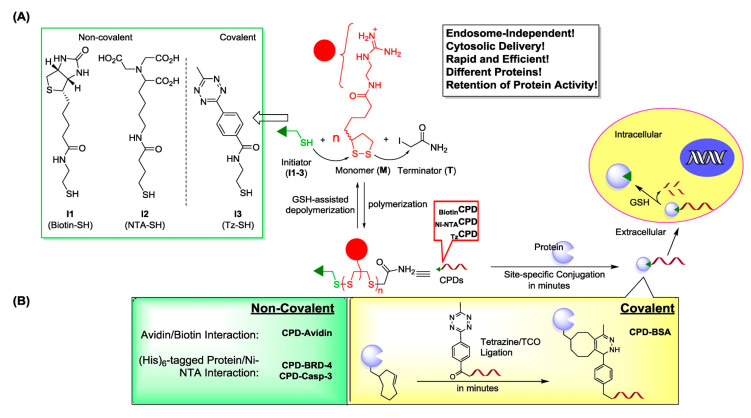
Overview of CPD-facilitated intracellular delivery of proteins (including antibodies) and native small-molecule drugs. (**A**) Newly developed initiators (I1/I2/I3), monomer (*M*), terminator (*T*), the polymerization/depolymerization process of CPDs, and the two-step approach for “conjugation” of protein cargos with CPDs. (**B**) Summary of non-covalent and covalent approaches for bioorthogonal attachment of CPDs to proteins. The highly efficient site-specific tetrazine-trans-cyclooctyne (TCO) ligation reaction is highlighted. Reproduced with permission from ref. [139]. Copyright 2015 American Chemical Society.

To overcome these challenges, Yao et al. further explored new protein-conjugation chemical methods that enable easy labeling of proteins with CPDs (Figure 6). Glycans present in mammalian proteins such as antibodies and glycosylated proteins can be modified to label the target proteins using post-translational modification (PTM) approaches. In particular, the traceless-tagging approach can be applied to modify both glycosylated and non-glycosylated proteins. Additionally, norbornene biorthogonal tags can be used to modify proteins, allowing self-degradation after CPD-enhanced cellular internalization, thereby releasing functional proteins. The norbornene bifunctional crosslinking agent (NBL), containing a disulfide bond, reacts with primary amines and can also connect CPD with norbornene through a biological orthogonal reaction. Similarly, PTM-labeled glycoproteins can be delivered and released into cells, followed by the rapid depolymerization of CPD while maintaining the native biological activity of the original protein with minimal impact on glycoproteins [139,140].

**Figure 7:**
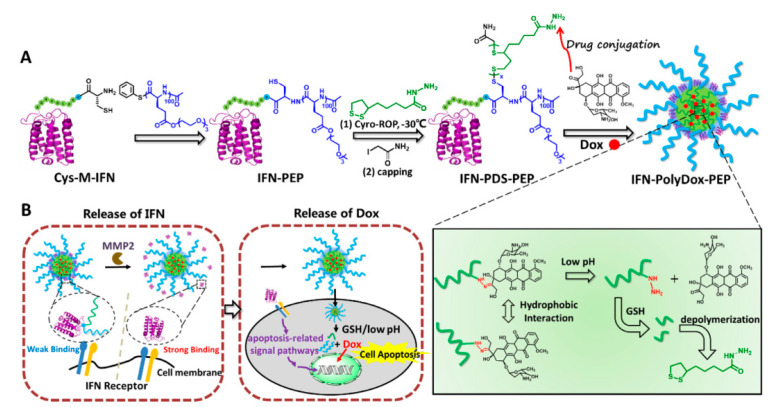
(**A**) Synthesis of IFN-PolyDox-PEP. (**B**) Cartoon illustration of the MMP-activatable, pH- and GSH-responsive release of IFN and Dox for enhanced chemo–protein combination therapy. Cyro-ROP, cryo ring-opening polymerization; Dox, doxorubicin; IFN, interferon-α2b; PDS, polydisulffde; MMP2, matrix metalloproteinase2; GSH, glutathione. Reproduced with permission from ref. [141]. Copyright 2021 Elsevier.

The cancer therapy approach, chemotherapy combined with the protein therapy, has a bright future. However, simple combination dosing or conventional preparation has not met people’s expectations yet. It is attractive and essential to develop novel cargos delivery strategies by taking advantage of the respective properties of protein and chemical drugs. Lu et al. designed and synthesized the multi-functional protein–drug-polymer conjugation based on GSH and acid responsiveness. The building blocks including interferon-α2b (IFN), polydisulfide reacted with Dox, and polypeptide (PEP) were integrated into a novel conjugation, termed IFN-polyDox-PEP. Especially, IFN-polyDox-PEP can self-assemble to form the stable nanoparticle with 122 nm with increasing the molar ratio of DOX and IFN to 103/1. Following aggregation into tumor and intracellular delivery of IFN-polyDox-PEP, it can release IFN because of conjugation disrupted by matrix metalloprotease in the tumor microenvironment, then complete the release of Dox upon GSH and acid stimulative response of CPDs. The developed IFN-polyDox-PEP nano preparation indicated better inhibition efficiency of tumor growth than that with the simple combination of Dox and IFN-polypeptide conjugation, which was on behalf of a potential strategy for synergistic therapy upon effective chemo–protein conjugation (Figure 7) [141].

## 5. Fluoropolymers for Cytosolic Protein Delivery

Polymers with specific compositions and topologies can be synthesized using various methods such as atom transfer radical polymerization, reversible addition-fragmentation chain transfer polymerization, and ring-opening metathesis polymerization. Additionally, these polymers can be easily modified with functional ligands for specific purposes and combined with degradable monomers or backbones for safety. Notably, cationic polymers showed great potential in cellular uptake and endosomal escape. The main challenge in this field is to produce stable complexes of cargo proteins using cationic polymers. These polymers are commonly used as carriers in gene delivery, as they bind and condense nucleic acids such as plasmid DNA and siRNA into nanoparticles due to their interaction with the negatively charged nucleic acids [142]. However, this approach is not suitable for proteins, as their net charge depends on both isoelectric point (pI) and the pH of their environment. Even for those proteins with low pI values, the number of net negative charges is not enough for the cationic polymers to form stable complexes. Stability of complexes is often compromised due to the presence of salts, polyanions, and phospholipids in physiological conditions. This is why there are numerous cationic polymers developed for gene delivery, but only a few for cytosolic protein delivery [15,143]. To address this, it is necessary to increase the binding affinity between the polymers and proteins, and/or reduce the repulsion between cationic polymers in the complexes. To this end, functional ligands can be attached to cationic polymers to form a stable complex with the cargo proteins [144,145]. Fluoropolymers are applied to construct amphiphilic polymers with a series of characteristics, which are used for intracellular proteins delivery [146,147,148]. Among them, the main reason for the obvious difference between fluoropolymer and hydrocarbon amphiphilic polymer is the hydrophobicity and lipophobic of fluoroalkane chain [149,150,151,152]. Therefore, in this review, we mainly discussed fluoropolymers-based delivery systems. First of all, fluoropolymers have self-assembly behavior in aqueous solution, which makes it possible for fluoropolymers and proteins to co-assemble to form stable complexes [153,154,155]. Secondly, the fluoropolymer has excellent efficient cell membrane permeability, and the hydrophobicity effect of fluoroalkyl chain improves the binding affinity of cell membrane [156]. However, in the process of endocytosis or direct membrane penetration, the lipophobic of fluoroalkyl chain can avoid the fusion of amphiphilic polymers and phospholipids [157]. Fluorinated cationic polymers improved the efficiency of various aspects in gene transportation, such as loading nucleic acid fragment ability, blood circulation, cell uptake, and endo/lysosomal escape [158,159,160,161,162,163]. Guan et al. successfully developed a bola small molecule [164]. The two ends of this small molecule are hydrophilic groups composed of lysine, histidine, and tryptophan, and the middle is a fluoroalkyl chain. The small molecule self-assembles with siRNA in aqueous solution to form nanoparticles, which is used for targeted gene therapy of adipocytes in adipose tissue with remarkable effect. The results showed that nanomaterials could rapidly release siRNA in adipocytes and hepatocytes, thus inducing 70% GAPDH gene knockout.

**Figure 8:**
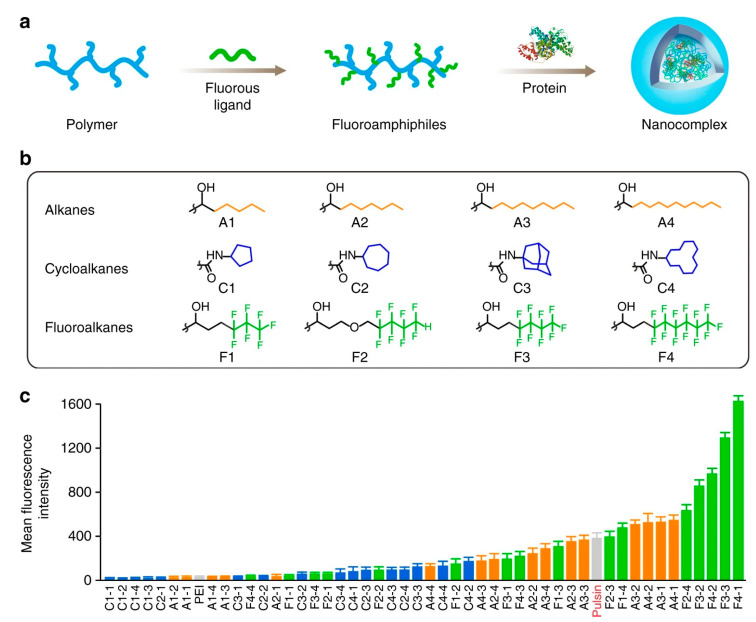
Fluoroamphiphiles for cytosolic protein delivery. (**a**) Co-assembly of fluoroamphiphiles and proteins. (**b**) Structures of hydrophobic substituents coupled to PEI. A1-A4 alkanes, C1-C4 cycloalkanes, F1–F4 fluoroalkanes. (**c**) Mean fluorescence intensity of cells transfected with nanocomplexes after Trypan Blue treatment. Reproduced with permission from ref. [157]. Copyright 2018 Nature.

Furthermore, fluoropolymers will bring new inspiration to the process of intracellular proteins delivery. To demonstrate this speculation, Cheng et al. synthesized a series of diverse fluoropolymers, which were synthesized by grafting fluoroalkane chains with different lengths and densities onto polyethyleneimine. Compared with commercial reagents, eleven kinds of polymers have better effects, seven of which are fluoropolymers (Figure 8). It is exciting that fluoropolymer F4-1 can efficiently deliver bovine serum protein into the cell. However, the hydrocarbon chain analogue without fluorineA4-1 could not promote the efficiency of intracellular proteins delivery. Fluoropolymers can promote the intracellular protein delivery efficiency compared to non-fluoropolymers, which should be ascribed to the role of fluorine in fluoropolymers [157]. More specifically, fluoropolymers are mixed with proteins to form uniform and relatively small nanoparticles. The influence of nanoparticles formed by fluoropolymer on the protein activity is relatively small, which is attributed to the fact that the fluorosis chain can not only resist fouling, but also effectively enhance cell membrane penetration efficiency. In addition, fluoropolymers exhibit lower toxicity than polymers without fluorine. These characteristics explain why fluoropolymers have relatively high efficiency in intracellular protein delivery.

For fluoropolymers, longer fluoroalkane chains and higher grafting ratio will improve the efficiency of cytosolic protein delivery [165,166,167]. However, too much fluorine in the polymer can also lead to the failure of protein intracellular delivery. Because, in the absence of protein, fluoropolymers will self-assemble to form stable vesicles. Therefore, polymers with different length of fluoroalkane chains and degrees of fluorination should be carefully evaluated and screened to achieve the best polymer. Although the screened fluoropolymers generally have a positive charge, they can enhance the efficiency of cytosolic anionic and cationic biomolecules delivery in different cell lines. The fluoropolymer can combine with the negatively charged groups of proteins with high pI value through electrostatic interactions, then further assemble to form nanoparticles through fluorophobic effect among fluoroalkane chains.

**Figure 9:**
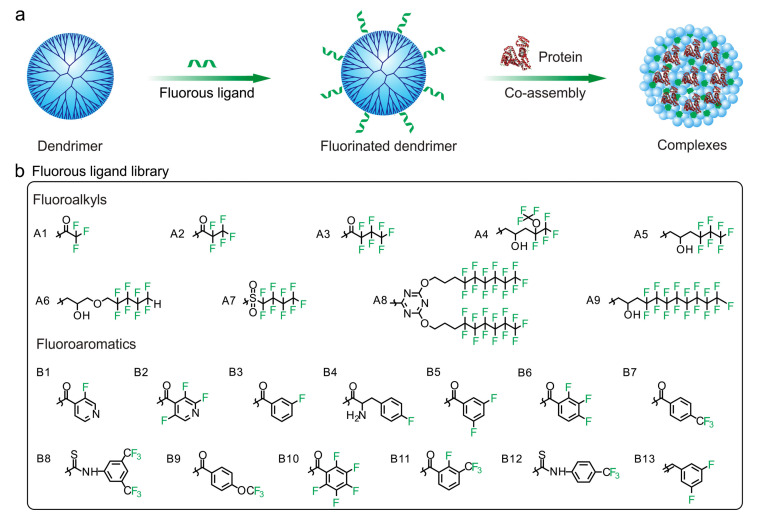
Screening of efficient polymers for intracellular protein delivery in a library of fluorinated dendrimers. (**a**) Self-assembly of fluorinated dendrimers and the proposed structure of polymer/protein complexes. (**b**) Structures of the conjugated fluorous ligands on dendrimer surface. A1-A9: fluoroalkyls; B1-B13: fluoroaromatics. Reproduced with permission from ref. [97]. Copyright 2018 Elsevier.

Recently, Liu et al. developed fluorocarbon-modified chitosan (FCS) for efficient delivery of various therapeutic proteins, such as immune checkpoint blockade antibodies. The results showed that at a 5-fold dose oral delivery of anti-programmed cell death protein-1, or its combination with anti-cytotoxic T-lymphocyte antigen 4 using this method, could achieve comparable antitumor therapeutic responses to those administered that administrated by intravenous injection of corresponding free antibodies in various types of tumor models [167]. In addition, Cheng et al. synthesized and developed a library of fluorine modified polymers for cytosolic cargoes delivery (Figure 9). The optimal fluoropolymer A6-2 without cytotoxicity could enhance the intracellular delivery efficiency of proteins such as β-galactosidase, bovine serum albumin (BSA), a cyclic decapeptide, and saporin. Moreover, the transported proteins and peptides remained native bioactivity after delivery and release in the cytosol. Because of the poor cell membrane permeability of saporin, it has little cytotoxicity on cancer cells. The A6-2 inefficiently deliver the saporin into the cytosol, which led to limited cell apoptosis. Compared to the A6-2/saporin with boring IC_50_ value of 13.2 nm, the hyaluronic acid coated A6-2/saporin with promising IC_50_ value of 3.3 nm could contribute to notable cell apoptosis. The prepared nanomaterials can transport saporin into the tumor tissue and efficiently inhibit the growth of tumors in vivo [97].

## 6. Challenges and Future Directions

In recent years, researchers developed various strategies to investigate a cytosolic protein delivery system, which is biocompatible, biodegradable, and dynamically functionalized. Meanwhile, these nanocarriers showed great promise for facilitating the current and next generations of treatments. Nevertheless, there still remain quite a few challenges including the following points:

- Once the protein structure was changed, and its therapeutic function would be lost. So, some barriers still need to be overcome during the preparation of proteins loaded nanocarriers, either covalent or noncovalent strategies. The development of nanocarriers in organic solvents could alter the structure of protein drugs.
- The materials chose to formulate the platform must be considered cell amicable. Most of these materials were validated in vitro and verified for biocompatibility and nontoxicity on various cells, which may not precisely predict the in vivo toxicity. Hence, there is a need to examine the in vivo results of exposure to nanomaterials before loading any therapeutic proteins, especially for materials that are not biodegradable.
- The mechanism of elimination and excretion of nanocarriers should be considered seriously. Because of many of them are resistant to elimination routes, for the large size and higher chemical stability. For example, semiconductor quantum dots injected into mice could remain intact over 2 years in mouse tissues.
- Though many of nanocarriers could be endocytosed into the endosomes, it is difficult for them to escape into the cytosol. High efficiency of endosomal escape is still a challenge for nanocarriers.
- The mechanism of the endocytosis-independent uptake of NPs summarized in this review was established by bioimaging, co-localization, and endocytosis inhibition to delicate endocytic efficiency of the nanocarriers. We are still convinced that the CPD technologies hold vast potentials either in routine laboratory or future clinical practices. However, there may be anxiety about the haptenization of cellular proteins and undesirable immune responses to neoantigen for the rearrangement of disulfide.
- Therapeutic protein drug delivery mediated by electrostatic interaction may lead to incomplete drug release, resulting in lower treatment efficiency. Therefore, the next-generation of delivery carriers needs to further optimize the impact of the carrier’s charge.
- Most studies cited in this review often used a mouse model to verify the biological activities of proteins loaded by nanocarriers, whether these therapeutic protein drugs showed biological activities after being delivered into targets in clinical trials need to be further verified. Although some of the protein drugs were used for clinical therapy, the protein-based nanomedicine research is still at an early stage of development and meets various challenges.

So far, several potential strategies were mentioned to develop protein-loaded nanocarriers aimed at enhancing drug encapsulation and improving endosomal escape efficiency of the protein drugs. Moreover, the symbiosis of diverse materials break new opportunities for protein-loaded multifunctional nanocarriers. For instance, CPD-based technologies gain significant benefits from their endocytosis-independent. In summary, the potential for protein-loaded nanocarriers in this field is tremendous. Based on current study advances and clinical trials, degradable backbones and increased endosomal escape efficiency are two of the most favorable structural properties for the future development of nanocarriers. As well as safety and potency, next-generation protein-loaded nanocarriers with extra functionalities such as targeting and immunomodulation will be important for specific applications. Although, more and more novel biomaterials or delivery vehicles are being used in this exhilarating field, there are still vast opportunities for strategies optimization and innovation to enable the broader delivery of therapeutic drugs.

## Data Availability

Not applicable.
